# The effects of roadway characteristics on farm equipment crashes: a geographic information systems approach

**DOI:** 10.1186/s40621-016-0096-1

**Published:** 2016-12-20

**Authors:** Mitchell Greenan, Maisha Toussaint, Corinne Peek-Asa, Diane Rohlman, Marizen R. Ramirez

**Affiliations:** 1 University of Iowa Injury Prevention Research Center, Department of Occupational and Environmental Health, University of Iowa College of Public Health, Iowa City, IA 52242 USA; 2 Department of Occupational and Environmental Health, Rm S318, University of Iowa College of Public Health, Iowa City, IA 52242 USA

**Keywords:** Traffic safety, Agricultural vehicles, Transportation, Road crashes

## Abstract

**Background:**

Tractors and other slow-moving self-propelled farm equipment are often used on public roadway to transfer goods from the farm to a market or distributer. Increased roadway exposure has led to a growing concern on the occurrence of farm equipment crashes. This study aims to compare characteristics of road segments with farm equipment crashes to road segments without farm equipment crashes in the state of Iowa.

**Methods:**

Data were obtained from the Iowa Department of Transportation from 2005 to 2011 on all crashes involving farm equipment, and features of all Iowa roadways. Geographic Information Systems (GIS) was used to identify geospatial features, such as road type, speed limit, traffic volume surface type, road and shoulder width of where a crash occurred. Logistic regression models were used to measure the associations between road characteristics and the occurrence of farm equipment crashes. Crude and adjusted odds ratios and 95% confidence intervals were reported.

**Results:**

A total of 1371 farm equipment crashes were reported in Iowa over the 6-year period and geocoded onto a street location. As traffic volume increased, the odds of a crash occurring also increased. Roadways with posted speed limits between 50 and 60 mph were associated with a higher odds of having crashes on them compared to roadways with speeds less than 35 mph (OR = 8.05, 95% CI: 6.59–9.84). Iowa routes (OR = 5.98, 95% CI: 4.97–7.20) had the highest odds of having crashes compared to local routes. Increased road width (OR = 0.90, 95% CI: 0.86–0.94) was associated with a 10% decrease in the odds of a crash.

**Conclusions:**

Higher traffic volume, higher posted speed limits, road type, and smaller road widths were associated with the occurrence of farm equipment crashes. Findings from this study can be used to guide policy to improve roadway design and conditions for all road users.

**Electronic supplementary material:**

The online version of this article (doi:10.1186/s40621-016-0096-1) contains supplementary material, which is available to authorized users.

## Background

In the United States, transportation-related incidents accounted for half of all agricultural worker fatalities in 2014, and of these incidents approximately 27% were due to crashes occurring on public roadways (Bureau of Labor Statistics U.S. Departement of Labor 2015). According to the Iowa Department of Transportation (IDOT) crash reports from 2004 to 2014, 2108 farm equipment crashes occurred on public roadways that resulted in 660 injuries and 79 fatalities (Iowa Department of Transportation 2015). Operating tractors and other slow-moving farm equipment on public roadways places agricultural workers at risk for collisions (Gerberich et al. 1996) and injuries. However, farm equipment crashes are not only an occupational hazard but also pose a significant threat to non-agriculture road users. Over a 10-year period, approximately 82% of farm equipment crashes involved non-farm vehicles, and among those resulting in at least one driver injury, drivers of non-farm vehicles were five times more likely to be injured than the farm equipment operators (Peek-Asa et al. 2007), demonstrating the need or urgency to develop and implement preventive measures.

Due to the severity of farm equipment crashes on public roadways, federal and state laws have been implemented but often require action with a focus on and at the expense of farmers. The mandatory use of appropriate equipment, lighting and vehicular signage to improve the safety and conspicuity of farm equipment on the roadway has been implemented in several states including Iowa, but enforcement is lacking (Committee on Agricultural Safety and Health Research and Extension 2009). Despite the fact that at least 88% of farmers reported lighting and marking their farm equipment according to regulatory standards (Luginbuhl et al. 2003), lack of visible signage and below-standard lighting remains a concern (Committee on Agricultural Safety and Health Research and Extension 2009; Kinzenbaw 2008).

State lighting and marking laws require individual behavior change on the part of the farm equipment operator. However, approaches to transportation safety research focus instead on identifying dynamic characteristics of the environment or the infrastructure of the roadway that predict the risk of a crash (Hadi et al. 1995; Karlaftis & Golias 2002). Ultimately, environmental infrastructure research can identify improvements in roadway design that can lead to significant reductions in the rate or severity of crashes. Recent roadway-based crash prediction models have consistently found that traffic volume (Hadi et al. 1995; Karlaftis & Golias 2002; Ackaah & Salifu 2011) was a significant predictor of motor vehicle crashes. Other road characteristics such as lane width, serviceability index (road quality), access control, pavement type (Karlaftis & Golias 2002), increased road segment length, terrain type (Ackaah & Salifu 2011), and shoulder width (Hadi et al. 1995) were also found to be significantly associated with the occurrence of a crash across studies. While these findings have been extremely important to the fields of transportation safety and public health, few studies have assessed the role of road characteristics on the risk of farm equipment crashes.

The few agriculture transportation-based studies report that farm equipment crashes frequently occur on roads with greater traffic volume (Costello et al. 2009), unpaved roads (Gerberich et al. 1996), roads in urban zip codes (Harland et al. 2014), two-lanes or county highways, and on roads with 55 mph posted speed limits (Gerberich et al. 1996; Peek-Asa et al. 2007; Gkritza et al. 2010). Although these studies have contributed to our knowledge concerning the challenges of operating farm equipment on roadways, there are important limitations to consider. One important limitation in crash report analyses is the lack of exposure information, which is necessary to estimate crash risk. To address this limitation, we analyzed farm equipment crash risks at the road-segment level and used Geographic Information Systems (GIS) to identify spatial attributes of road segments with and without crashes. The aim of this study was to investigate roadway characteristics that are associated with farm equipment crashes on Iowa roadways. We compared characteristics of road segments where farm equipment crashes occurred to road segments where there were no farm equipment crashes.

## Methods

### Data source

Data on all crashes involving farm equipment on public roads in Iowa from 2005 to 2011 were obtained from the IDOT. Farm equipment was defined as vehicles designed specifically for agricultural operation (2014) such as combines, farm tractors, fertilizers, feeders, towed grain carts, and wagons. Although pick-up trucks can be used as farm equipment, we excluded them because we could not verify the purpose of use. From the crash data that include crash latitude and longitude, each reported crash was geocoded using GIS to provide a geographic view or visualization of where farm equipment crashes occurred throughout the state. Each crash was then linked to a road segment. Of the 1401 farm equipment crashes, 12 were coded as passenger vehicles based on vehicle identification numbers, and 18 records were missing address-based data and were excluded resulting in a final sample size of 1371. Rather than using the crash as the unit of analysis, we used the road segment on which the crash occurred. Road segment data were provided by the Environmental Services Research Institute (ESRI). Roads were demarcated into segments each time any of the road characteristics (e.g., type of road, speed, and road width) changed (see Appendix). The IDOT road network spatial dataset created in 2007 of Iowa roads (primary, secondary, and municipal roads) containing information describing the road segments was used for this study. All study procedures were reviewed and approved by the University of Iowa Internal Review Board.

### Study population

The original dataset consisted of 324,769 road segments collected by the IDOT. Approximately two percent of the original dataset had missing data for speed limit (*n* = 5064), road width (*n* = 4139) and surface type (*n* = 3871). The removal of these records resulted in a final sample at 319,705 road segments.

### Study variables

The outcome, road segments where a farm equipment crash occurred, was defined as a public Iowa roadway where a vehicular crash involving at least one piece of farm equipment occurred. The road segment characteristics of interest were traffic volume, road width, shoulder width, speed limit, road type, and surface type. Traffic volume was assigned based on actual and estimated annual average daily traffic (AADT), which was calculated by the IDOT as the number of motor vehicles driven on a given road segment per day. Traffic volume was either physically counted or estimated through a spatial extrapolation method used by the IDOT (AADT was assigned based on surrounding AADT values or AADT values of similar road types and numbers of lanes). Road width was defined as the total width measured in feet, excluding shoulders. Shoulder width, measured in feet, was defined as the sum of the left and right shoulders of a road. Speed limit was defined as the lowest posted speed limit per road segment. This variable was recoded into four categories: <35, 35–45, 50–60, and 60+. The IDOT classified road type using five road system classifications, and each road segment was grouped into one of the following categories: Interstate, US route, Iowa route, farm to market route, or local route. Farm to market routes are public roads meant specifically for the transport of goods from farms to towns or cities. Local routes are either 25 mph residential roads or 55 mph rural roads. The IDOT classified surface type into 42 categories which were dichotomized into paved (e.g. asphalt, concrete, or brick) or unpaved (e.g. gravel or stone without admixture, grade and drained earth without borrow topping - no shoulder, and unknown).

### Analysis

Cases were defined as road segments that had at least one farm equipment crash. Univariate analyses were used to report the distribution of each variable for all Iowa roads and stratified by crash status. Chi-square or t-test was conducted to examine the relationship between exposure variables and the occurrence of a crash. Collinearity between all covariates was assessed using Pearson correlation. Cochran-Armitage trend test was used to assess a trend in the proportion of farm equipment crashes across categories for traffic volume. Logistic regression models were used to measure the risk of a crash by estimating odds ratios and 95% confidence intervals. All statistical analyses were conducted using SAS 9.3 for Windows.

## Results

**Fig. 1:**
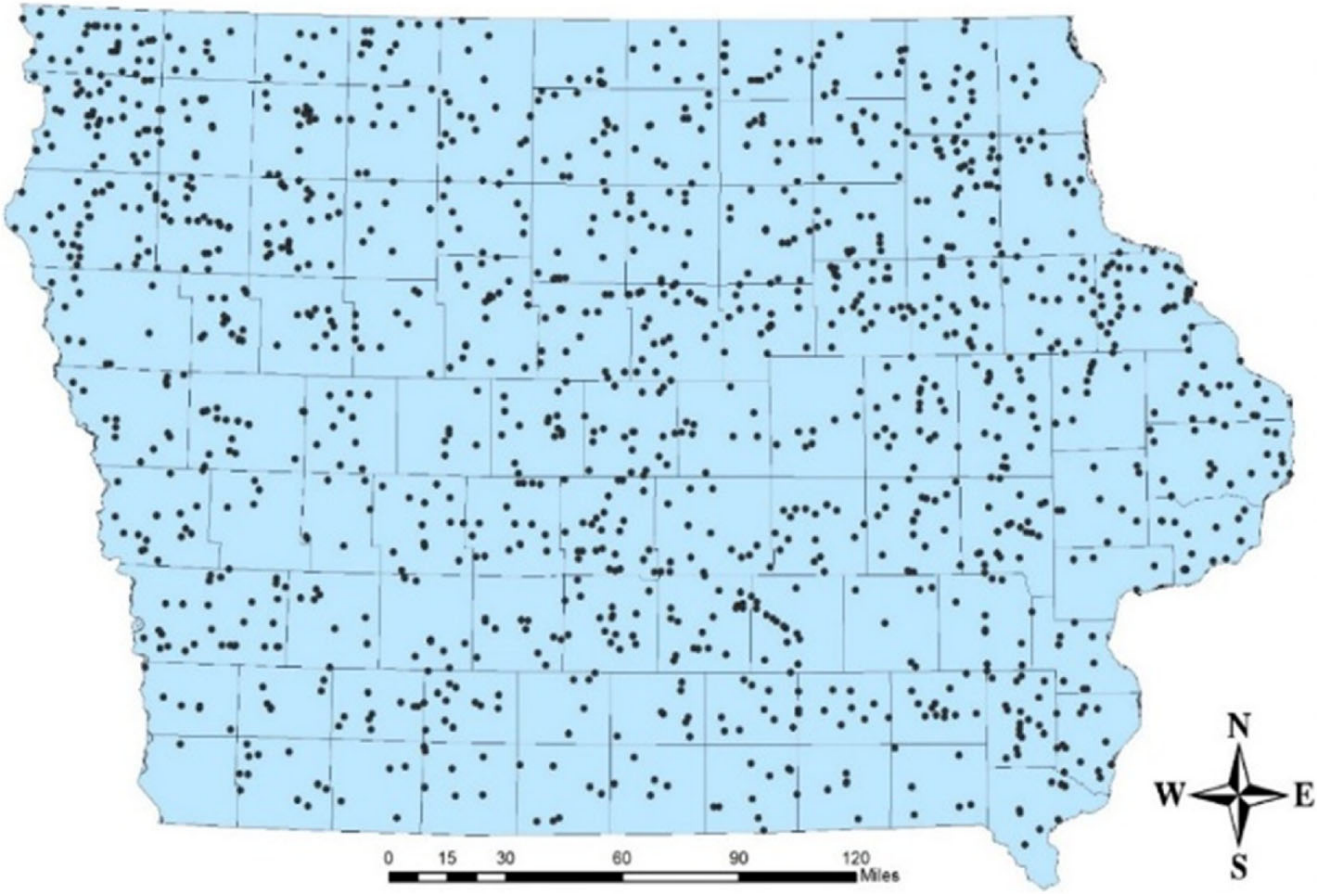
The distribution of the 1371 farm equipment crashes across Iowa using GIS

There was a total of 1371 farm equipment crashes reported from 2005 to 2011, and these occurred on 1337 road segments, less than one percent of Iowa’s 319,705 road segments. An illustration on the distribution of farm equipment crashes occurring throughout the state of Iowa was created using GIS (Fig. 1). Crashes were randomly dispersed across the state and not confined to an isolated geographic area. The highest percentage of crashes occurred on road segments with an average annual daily traffic count of at least 1251 vehicles (33%), with posted speed limits between 50 and 60 mph (79%) and on paved roads (70%) (Table 1). The majority of farm equipment crashes occurred on farm to market routes (43%), followed by 31% on local routes.

**Table 1:** Roadway characteristics of all Iowa road segments stratified by crash status. N(%)*

	Road segments		
	Without a Crash (*n* = 318,368)	With a Crash (*n* = 1,337)	All Iowa Roads (*n* = 319,705)
Road characteristics			
Traffic volume^a^			
0 – 30	68,068 (21.4)	123 (9.2)	68,191 (21.3)
31 – 101	63,165 (19.8)	220 (16.5)	63,385 (19.8)
102 – 360	62, 806 (19.7)	192 (14.4)	62,998 (19.7)
361 – 1250	62,283 (19.6)	367 (27.5)	62,650 (19.6)
1251+	62,046 (19.5)	435 (32.5)	62,481 (19.5)
Speed limit (mph)			
< 35	118,946 (37.4)	156 (11.7)	119,102 (37.3)
35 – 45	18,057 (5.7)	85 (6.4)	18,142 (5.7)
50 – 60	173,207 (2.6)	1053 (78.8)	174,260 (54.5)
65+	8,158 (2.6)	43 (3.2)	8,201 (2.6)
Road type			
Interstate	5,323 (1.7)	6 (0.5)	5,329 (1.7)
US Route	18,265 (5.7)	159 (11.9)	18,424 (5.8)
Iowa Route	17,425 (5.5)	191 (14.3)	17,616 (5.5)
FTM Route^b^	62,962 (19.8)	569 (42.6)	63,531 (19.9)
Local Route	214,393 (64.3)	412 (30.8)	214,805 (67.2)
Surface type			
Paved	190,576 (59.9)	930 (69.6)	191,504 (59.9)
Unpaved	127,794 (40.1)	407 (30.4)	128,201 (40.1)
Road width			
mean(sd)	25.2 (7.3)	24.7 (5.8)	25.2 (7.3)
Shoulder width			
mean(sd)	4.2 (5.6)	7.4 (6.2)	4.2 (5.6)

^a^Average Annual Daily Traffic (Total annual traffic volume/365)

^b^Farm to market route

*P < 0.01 for all variables

We found a significant increasing trend in the proportion of farm equipment crashes for traffic volume (Z = 15.82, *p* < 0.0001) (not tabled). As traffic volume increased, the proportion of farm equipment crashes also increased. Road segments with traffic volume of 361–1250 or 1251 or more vehicles per day had 7.43 (95% CI: 5.90–9.34) or 7.00 (95% CI: 5.38–8.85) times the odds of having a farm equipment crash compared to road segments with traffic volume of 30 or less vehicles per day, respectively (Table 2). Road segments with posted speed limits in the 50–60 mph category had eight times the odds of a crash compared with road segments with less than 35 mph speed limits (95% CI: 6.59–9.84). US routes (OR = 4.86, 95% CI: 3.99–5.91), Iowa routes (OR = 5.98, 95% CI: 4.97–7.20), and farm to market routes (OR = 4.67, 95% CI: 4.11–5.32) had higher odds of farm equipment crashes compared to local routes, respectively. For every five-foot increase in roadway width, the odds of a crash decreased by 10% (OR = 0.90, CI: 0.86–0.94). For every five-foot increase in shoulder width, the odds of a crash decreased by 6% (OR = 0.94, CI: 0.89–1.00). However, this estimate was marginally statistically significant.

**Table 2:** Odds of farm equipment crashes occurring on Iowa road segments calculated using Logistic Regression

	Farm equipment crash	
	cOR (95% CI)^a^	aOR (95% CI)^b^
Road characteristics		
Traffic volume^c^		
0–30	Ref	Ref
31–101	**1.93 (1.54–2.40)**	**2.00 (1.60–2.49)**
102–360	**1.69 (1.35–2.12)**	**3.76 (2.97–4.77)**
361–1250	**3.26 (2.66–4.00)**	**7.43 (5.90–9.34)**
1251+	**3.88 (3.17–4.74)**	**7.00 (5.38–8.85)**
Speed limit (mph)^d^		
< 35	Ref	Ref
35–45	**3.59 (2.75–4.68)**	**3.03 (2.30–3.99)**
50–60	**4.64 (3.92–5.49)**	**8.05 (6.59–9.84)**
65+	**4.02 (2.87–5.64)**	**3.52 (2.42–5.14)**
Road type^e^		
Interstate	0.59 (0.26–1.31)	0.60 (0.27–1.35)
US route	**4.53 (3.77–5.44)**	**4.86 (3.99–5.91)**
Iowa route	**5.70 (4.80–6.78)**	**5.98 (4.97–7.20)**
FTM route^f^	**4.70 (4.14–5.34)**	**4.67 (4.11–5.32)**
Local route	Ref	Ref
Surface type		
Paved	Ref	Ref
Unpaved	0.65 (0.58–0.73)	0.97 (0.86–1.11)
Road width^g^		
mean(sd)	**0.95 (0.91–0.99)**	**0.90 (0.86–0.94)**
Shoulder width^g^		
mean(sd)	**1.44 (1.39–1.49)**	**0.94 (0.89–1.00)**

^a^Crude odds ratios and 95% confidence intervals

^b^Adjusted odds ratios and 95% confidence intervals; bolded estimates were significant at the α=0.05 level.

^c^Average Annual Daily Traffic (Total annual traffic volume/365)

^d^Model mutually controls for Speed Limit, Traffic Volume, and Shoulder Width

^e^Model mutually controls for Road Type, Surface Type and Road Width

^f^Farm to market route

^g^Unit: 5 feet

## Discussion

This is the first retrospective cohort study of farm equipment crashes using road segments as the unit of analysis. As such, this study allows for a robust evaluation of roadway characteristics that increase the risk of a farm equipment crash. We observed a positive dose response relationship between traffic volume and the risk of a crash: as the annual average number of vehicles traveled per day increased, the risk of farm equipment crashes also increased. This suggests that when a piece of farm equipment is on the road with a greater number of vehicles, the increased volume leads to a greater chance of being involved in a crash. Prior research has consistently found that high traffic volume increases crash risk in studies that have not focused specifically on special vehicles like farm equipment (Karlaftis & Golias 2002; Ackaah & Salifu 2011; Wang et al. 2009). Wider lane width proved to be a significant protective factor against crashes potentially by decreasing the proximity between farm equipment and opposing traffic (Karlaftis & Golias 2002).

Wider lane and shoulder width were also protective factors in this analysis, associated with at least a 6% decrease risk in crashing (Hadi et al. 1995; Karlaftis & Golias 2002). The size of farm equipment ranges between 10 and 25-foot-wide, which is much wider than the standard 12-foot-wide lane (U.S. Interstate Highway System). As a result farm equipment may occupy multiple lanes (Committee on Agricultural Safety and Health Research and Extension 2009). Among citations received by farm equipment operators, 13% were cited for being left of center, suggesting further that standard roadway lanes are unable to accommodate large farm equipment (Luginbuhl et al. 2003). Furthermore, smaller lanes and lack of shoulders may contribute to farm equipment or other vehicles running off the road, resulting in both collision and non-collision crashes. Lack of road width to maneuver around slow-moving farm equipment can be problematic for drivers of farm equipment and passenger vehicles when navigating the roadways.

During an attempt to pass such a large structure, drivers of non-farm vehicles must enter the opposing lane to determine if it is safe to pass, which may inevitably result in a crash (Kinzenbaw 2008). Furthermore, the frequent presence of towed implemented behind the farm equipment complicates visualization and increases time required to pass (Committee on Agricultural Safety and Health Research and Extension 2009). The validity of these scenarios is supported by the fact that passing collisions are the second most common type of farm equipment collisions (Kinzenbaw 2008; Gkritza et al. 2010) and are usually severe crashes. Of farm equipment crashes that resulted in injury, 22% of non-farm vehicles were passing the farm equipment (Peek-Asa et al. 2007). With wider shoulders, farm equipment are not only able to pull over in the event of a passing to prevent sideswipes but are also able to drive on the shoulder while staying within the lane.

Road type was also a significant contributor to farm equipment crashes. Farm to market, Iowa and US routes had higher risk of a crash, while interstate roads had the lowest risk. The increased risk observed could be indicative of the increased presence of farm equipment on these types of roads compared with other roads. The inverse may explain the lower risk of farm equipment crashes observed on interstates compared to local routes. In fact, the operation of farm equipment on interstate roads is illegal according Iowa state laws (2014).

Another explanation for the increased risk of crashes particularly on farm to market roads is urban sprawl, which occurs as individuals are widely dispersed into less occupied or dense areas, resulting in long commutes and high traffic exposure (Ewing 1997). As a result, roads that were primarily used for agriculture purposes are now being used more by non-farm vehicles during their commute (Costello et al. 2009). The interaction between suburban or urban motorists and farm equipment operators becomes problematic particularly for drivers unfamiliar with the challenges of sharing the roadway with farm equipment due to its large size, slow speed, and limited maneuverability.

Our study also found that roadways with posted speed limits in the 50–60 mph category were at greater risk for farm equipment crashes. Motorists driving at higher speeds have less time to react to a slow-moving vehicle creating significant challenges for approaching vehicles due to the rate of approach (Kinzenbaw 2008; Jaarsma & De Vries 2014). To put this into perspective, a passenger vehicle traveling 50–60 mph may potentially close a 400-foot gap with farm equipment moving at a speed of less than 25 mph in less than 10 s (Schwab 2013). All in 10 s, a driver must analyze the situation to identify the potential hazard and determine an adequate reaction, which may not be sufficient time to prevent a crash. The substantial difference in speed provides a plausible explanation for rear-end collisions (20–62%) being the most frequent type of farm equipment crashes in prior studies (Gerberich et al. 1996; Gkritza et al. 2010) and the high number of “failure to reduce speed” violations received by non-farm vehicle drivers (Hughes & Rodgman 2000).

Prior research suggests that farmers are highly aware of the safety hazards on high-speed roads. Seventy-seven percent of farmers in North Carolina reported feeling unsafe while driving farm equipment on the roadway due to the speed of other motorists (Luginbuhl et al. 2003). Lack of education on the recognition of the slow-moving vehicle (SMV) emblem, particularly among urban/suburban motorists, was suggested by farmers as the main contributing factor to crashes. To corroborate this statement, a survey conducted by (Garvey 2003) found that less than 30% of 18–84 year-old drivers were able to correctly state the purpose of the SMV emblem. Two approaches to decrease risk for farm equipment crashes could include driver education to raise awareness of safe practices for sharing the road with farm equipment, or to lower the speed limit of rural roads, specifically on farm to market and local routes. Over the last decade, academic organizations and government agencies have disseminated safe driving information through videos, driver’s education manuals, and pamphlets throughout the state on making safer decisions in high traffic areas where farm equipment are present (Kinzenbaw 2008; Iowa’s Center for Agricultural Safety and Health 2015). Findings from our study and others can contribute to educational efforts by identifying the environmental or road characteristics that are predictive of farm equipment collisions and providing insights on causes.

### Limitations

Crash data are subject to misclassification of farm equipment involved in a crash, since first responders at the scene are responsible for this categorization. For example, pick-up trucks, which may be used as farm equipment, may be classified as passenger vehicles or farm equipment, although they do not share the same risk factors as farm implements. We excluded pick-up trucks from the farm equipment category if make and model were available on reports. This exclusion and misclassification may have underestimated the effects observed in this study. Another limitation is the lack of roadway travel data specific to farm equipment, which limits the interpretation of our findings. For example, while traffic volume is available for each road segment, we do not know how many farm equipment travelled on a particular road segment, if at all. Unfortunately, traffic counts are not reported by vehicle type.

## Conclusion

Despite these limitations, our findings contribute to the traffic and agricultural literature in a number of substantive ways. We found that increased traffic volume, posted speed limits, and smaller roadway widths were all significantly associated with the occurrence of farm equipment crashes. Findings from this study can be used to effectively guide the development of policies that promote the education of roadway users on sharing the roadway with farm equipment and of engineering initiatives to design safer public roadways for all road users.

## Appendix

**Table 3:** Roadway Characteristics that Demarcate IDOT Road Segments

Change in divided roadway to non-divided roadway, or vice versa	
Significant change in outside shoulder width (0.6 meters or more)	
Significant change in median width (0.6 meters or more)	
Change in surface type	
Change in surface width	
Change from two-way to one-way street, or vice versa	
Section with “Y” in SPECIAL STUDY	
Change in surface condition rating (two points or more)	
Change in state functional classification	

## Additional file

Additional file 1: List of roadway characteristics that demarcate Iowa road segments. (XLSX 97076 kb)
